# Exploring physical activity experiences among children and adolescents during and beyond cancer treatment: a meta-synthesis of qualitative research

**DOI:** 10.1186/s12885-025-15490-1

**Published:** 2026-02-02

**Authors:** Amanda Wurz, Jenson Price, Madison Oser, Megan Filiatrault, Hannah Conlan, Sarah L. Grimshaw

**Affiliations:** 1 School of Kinesiology, Faculty of Health Sciences, University of the Fraser Valley, Chilliwack, BC Canada; 2 Faculty of Kinesiology, University of Calgary, Calgary, Canada; 3 School of Kinesiology and Health Studies, Faculty of Arts and Science, Queen’s University, Kingston, Canada; 4 Department of Occupational Science & Occupational Therapy, Faculty of Medicine, University of British Columbia, Vancouver, Canada; 5 Murdoch Children’s Research Institute, Cancer Therapies Group, Melbourne, Australia

**Keywords:** Pediatric oncology, Adolescent oncology, Exercise, Movement, Lived experiences, Supportive care, Survivorship, Systematic review

## Abstract

Children and adolescents diagnosed with cancer experience negative effects during and beyond treatment that span physical, psychosocial, and cognitive domains. Physical activity (PA) is a feasible, safe, and recommended strategy to mitigate many of the negative effects. As PA continues to gain attention as a positive health behaviour, systematic synthesis and integration of the accumulating body of qualitative research is necessary to gain a deeper and more nuanced understanding of this population’s experiences with PA and to support future intervention/program design and implementation efforts. The objectives of this meta-synthesis were to: (i) identify and describe findings from articles qualitatively exploring PA experiences among children and adolescents diagnosed with cancer, (ii) integrate, compare, and contrast findings to clarify patterns and develop an overarching narrative, and (iii) identify knowledge gaps to inform recommendations for future research and practice. A meta-study methodology was adopted, and three electronic databases were searched (initial search on May 19, 2023, search update on April 7, 2025). Two researchers screened citations against pre-defined eligibility criteria and extracted data from included articles. Reporting completeness was assessed using a standard qualitative reporting checklist and data were analyzed using meta-method, meta-theory, and meta-data analyses. Database searching identified 5061 unique references, of which 27 met eligibility criteria and were included herein (*n* = 798 participants; range = 3-128 participants). Reporting completeness ranged from ‘poorly reported’ to ‘well-reported’. Most articles were experimental (*n* = 14), few specified the qualitative methodology used (*n* = 8), and fewer reported adopting a guiding theory/model (*n* = 7). Interviews were the most common data collection approach (*n* = 20). Across articles, six themes (with additional subthemes) were identified and organized into two overarching categories to highlight dominant ideas: (i) PA experiences and (ii) influencing factors. Summarized data collectively highlighted the varied influences that can shape motivation for and engagement in PA, the complex interplay between cancer and PA experiences, and the range of potential benefits–spanning physical, emotional, and social domains–that PA may confer. Future efforts should prioritize methodological rigor, theoretical integration, and co-designed approaches to ensure that PA opportunities are accessible, meaningful, and feasible for this population.

**Supplementary Information:**

The online version contains supplementary material available at 10.1186/s12885-025-15490-1.

## Background

Cancer diagnosed among children and adolescents (ages 0–19; pediatric cancer) is relatively rare, with an estimated 400,000 diagnosed globally each year [1]. Though survival rates continue to increase, with nearly 85% of those diagnosed surviving ≥ 5 years and > 70% surviving ≥ 10 years [2], pediatric cancer remains a leading cause of morbidity (e.g., cardiopulmonary conditions), secondary cancers, and premature mortality [3–8]. Further, a number of side effects (e.g., nausea, pain, fatigue; [9–12]) are reported by children and adolescents during treatment and adverse physical, psychosocial, and cognitive effects are described by those following treatment [13–19]. The persistent and far-reaching nature of these challenges necessitates heightened attention to strategies that can mitigate the negative effects associated with pediatric cancer.

One strategy is physical activity (PA); defined as any bodily movement produced by skeletal muscles that results in energy output beyond resting [20]. PA has been shown to be doable during and beyond treatment, low-risk (i.e., minimal to no adverse events), and as possibly reducing the severity of some treatment-related side effects and improving a range of physical, psychosocial, and cognitive outcomes in the short- and long-term [21–27]. Greater PA has also been associated with reductions in morbidity, secondary cancers, and premature mortality [28]. These findings have been summarized in numerous systematic reviews and meta-analyses that collectively suggest PA is feasible, safe, and beneficial for children and adolescents diagnosed with cancer during (i.e., pediatric cancer patients) and beyond treatment (i.e., pediatric cancer survivors)(e.g [29–36]), and captured in guidelines recommending PA [37, 38]. Though the beneficial role of PA in pediatric cancer is clear, most pediatric cancer patients and survivors engage in significantly less PA than their healthy peers [39, 40], adherence to PA interventions is varied [41, 42], and few have sought to identify outcomes that are relevant to end users, including pediatric cancer patients and survivors [43]. Thus, while the body of evidence supports PA, important questions remain.

Qualitative methods (e.g., interviews, open-ended questions) may be an ideal approach to address these gaps and better understand reasons for low PA, barriers and facilitators to PA (including PA intervention) participation, and experiences with (and perceptions of) PA among pediatric cancer patients and survivors. Qualitative methods centre participants’ lived experiences, interpretation, explanation, and meaning of phenomena, leading to deeper and more nuanced understandings that consider important contextual information [44]. For example, Larsen et al., [45] conducted interviews with 63 pediatric cancer survivors (and their parents) and discovered the significant and pervasive negative impact of treatment-related long-term or late effects on PA. Participants elucidated the relationships between their reduced physical capacity and lack of opportunities, perceived ability gap between themselves and their peers, and motivation for PA. They went on to describe how these complex relationships were further influenced (both positively and negatively) by environmental, interpersonal, and individual factors. These findings provide deeper insight into factors that can be considered in PA intervention/program design to reduce barriers and optimize facilitators. Though this study (and others like it) are valuable, single qualitative studies are rarely used on their own to augment practice [46]. Like their quantitative counterparts, a systematic review and integration of findings from the collective body of qualitative evidence is critical. Yet few efforts have been made to identify or summarize available qualitative evidence. One notable exception was published by, Brown et al., [47] reporting a meta-synthesis of qualitative evidence related to barriers and facilitators to PA among pediatric cancer survivors. Eight articles were identified, and descriptive themes were mapped onto the 9 domains of the Theoretical Domains Framework. Beyond this article, focused on a specific subset of the pediatric cancer population, most available published reviews on the topic of PA in pediatric cancer have focused primarily on quantitative data. Further, where qualitative studies have been included, no meta-synthesis has been performed. Thus, the overarching purpose of this meta-synthesis was to identify, analyze, synthesize, and interpret qualitative findings related to PA experiences among pediatric cancer patients and survivors. Specific objectives were to: (i) identify and describe findings from articles qualitatively exploring pediatric cancer patients’ and survivors’ experiences with PA, (ii) integrate, compare, and contrast findings to clarify patterns in conclusions and develop an overarching narrative of experiences with PA among pediatric cancer patients and survivors, and (iii) identify knowledge gaps to inform recommendations for future research and practice.^1^

## Materials and methods

The protocol for this review was prospectively registered in PROSPERO (registration number: CRD42023432809). Guidance from Paterson et al. [46], was followed and work progressed sequentially. Reporting follows the enhancing transparency in reporting the synthesis of qualitative research (ENTREQ; [48]) reporting framework.

A relativist perspective [49] was adopted, which recognizes reality is subjective, context-dependent, and shaped by individual and cultural perspectives. The research team viewed knowledge as pluralistic and situated, emerging from diverse lived experiences and interpretations. Thus, the intention of this meta-synthesis was not to reconcile or privilege one perspective in the literature over another, but to document, respect, and amplify the diversity of voices and interpretations. The analysis was therefore shaped through the perspectives of the research team who hold a positive view of PA, have developed, delivered, and/or evaluated PA interventions in pediatric oncology (and other clinical populations), and have a professional commitment to building access to and engagement in PA-based supportive opportunities for pediatric cancer patients and survivors (and other clinical populations).

With respect to the sequential steps, first, research questions to guide this meta-synthesis were developed by four research team members (AW, JP, MO, HC) based on substantive content knowledge, qualitative meta-synthesis methodology expertise, and preliminary database searches. Next, 3 electronic databases were systematically searched: (i) Medical Literature Analysis and Retrieval System Online (MEDLINE), (ii) PsycINFO, and (iii) SPORTDiscus. The search strategy was developed collaboratively with research team members and a university librarian (DC) drawing on search strategies from previously published reviews (e.g [29–36]). MeSH terms and keywords covered the population (i.e., child, children, pediatric(s), adolescent(s), cancer, neoplasm(s), oncology, tumour) and terminology associated with PA (e.g., exercise, sports, yoga, physical, endurance, strength). No MeSH terms or keywords covering data collection methods were included. This was done to enhance the likelihood of identifying all published articles that explored pediatric cancer patients’ and survivors’ experiences with PA. Restrictions were placed on publication date (2000 onwards) and English language. Once the strategy was piloted and finalized in MEDLINE, it was translated to PsychINFO and SPORTDiscus. Adjustments were made as necessary, to reflect the varying syntax, indexing terms, and search functionalities of the databases while maintaining the core concepts and terms (see Supplementary File 1 for search strategies). The databases were searched May 19, 2023 and a search update was performed on April 7, 2025. Results from searches were exported to RIS format and uploaded to Covidence, a systematic review online platform to store, organize, and manage citations.

After the automatic removal of duplicate records by the Covidence software, three authors were trained (MO, HC, MF) and two independently screened the first 100 citations at the title/abstract level. They then met with a third author (AW) to confirm shared understanding of eligibility criteria and processes. This was repeated with an additional 100 citations. Following this, the two authors independently screened the remaining citations in two stages: (i) title/abstract and (ii) full-text. At each stage, articles were excluded if they did not meet the eligibility criteria outlined below. In cases of disagreement at both levels, reviewers discussed, and if needing further guidance, consulted with a third author (AW or JP). Cohen’s kappa was calculated to measure inter-coder agreement at both screening steps, and can be interpreted as follows: 0–0.20 = none, 0.21–0.39 = minimal, 0.40–0.59 = weak, 0.60–0.79 = moderate, 0.80–0.90 = strong, > 0.90 = excellent agreement [50].

Eligibility criteria were set a priori, and articles were included if they: (i) used any experimental study design (e.g., randomized controlled trials, non-randomized studies, interrupted time series, controlled before and after studies) evaluating PA or observational study design exploring PA, (ii) adopted qualitative or mixed methods approaches, provided pediatric cancer patients’ and/or survivors’ experiences with PA were captured via qualitative methods (e.g., interviews, open-ended questions), and (iii) included pediatric cancer patients and/or survivors, defined as: children and/or adolescents (< 19 years of age) during and beyond treatment (at any stage along the cancer trajectory either awaiting, undergoing, or post-treatment). In cases where some of the sample was > 19 years of age, the mean age of study participants was required to fall < 19 years of age. In instances where multiple perspectives were gathered (e.g., studies exploring pediatric cancer patients’ and survivors’ and their parents’ experiences), the mean age of children and adolescents diagnosed with cancer was required to fall < 19 years of age. Articles were excluded if a full-text was not available, if the report was not in English, if qualitative data and findings were not included within the results section, and/or when PA was not a topic/intervention within the article. Grey literature (e.g., conference abstracts/posters/proceedings, unpublished theses/dissertations), books, opinion pieces, and reviews were also excluded.

The following data were extracted using a template housed on Covidence: (i) study information (ie, authors, country of data collection, year of publication), (ii) study characteristics (ie, study design, sampling methods, sample size, methodology, data collection methods, analysis methods), (iii) sample characteristics (ie, age, percent girls/females, type of cancer(s), disease stage, timing), (iv) as applicable, reported PA characteristics (ie, dosage [frequency, length, duration], location, type, social setting, mode of delivery), (v) conceptual/theoretical approaches, and (vi) qualitative findings. Of note, in instances where some participants were > 19 years of age or in the case multiple perspectives were gathered (eg, studies exploring pediatric cancer patients’, survivors’, and their parents’ experiences with PA), only data pertaining to those meeting eligibility criteria (i.e., pediatric cancer patients and/or survivors) were extracted. If information presented in the primary article was unclear or missing, corresponding authors were contacted via email to obtain clarification or missing information (maximum 3 attempts). To ensure completeness and accuracy in extraction, after MO and HC/MF independently extracted data from included articles, JP conducted a validation check by randomly selecting 20% of the articles, independently extracting the data, and comparing the results to identify and resolve any discrepancies through team consensus.

Following the relativist perspective adopted [49], one author (JP) independently appraised the trustworthiness, theoretical considerations, and practical considerations of each study using the 21-item Standards for Reporting Qualitative Research (SRQR) checklist [51]. Specifically, JP assessed whether included articles reported the methodological components transparently in the title and abstract (*n* = 2 items), introduction (*n* = 2 items), methods (*n* = 11 items), results (*n* = 2 items), discussion (*n* = 2 items), and other (*n* = 2 items) sections of the article. Reporting completeness, assessed using the SRQR was not an eligibility criterion. Instead SRQR data were used to furnish the meta-method analysis and provide insight into elements of qualitative research that may not be commonly reported in this area to better identify gaps and inform recommendations for future research.

Data analysis included: (i) meta-method analysis, (ii) meta-theory analysis, and (iii) meta-data analysis. A descriptive synthesis was used for the analysis of the methods and theories, which enabled an overview of the methodological and theoretical landscape of included articles. A thematic synthesis approach [52] was adopted for the analysis of the qualitative data, which enabled the identification of common themes and subthemes across included articles. This dual approach was deemed well-suited for this meta-synthesis, wherein a broad scope was adopted due to the diverse methods and theories used and outcomes reported across articles. For the meta-method and meta-theory analyses, one author (JP) independently synthesized the methods used (i.e., study design, sampling technique, qualitative data collection procedures and analytical approach) and theories adopted (i.e., research paradigm, conceptual and/or theoretical approach adopted). Frequencies, percentages, averages and standard deviations, and medians and interquartile ranges were calculated when appropriate to summarize specific characteristics of the methods and theories used across studies.

For the meta-data analysis, two authors (AW, SG) reviewed the extracted qualitative findings several times to familiarize themselves with the data. Next, AW and SG uploaded included articles into NVivo, and then independently coded the qualitative data (i.e., direct participant quotes) within each article using a hybrid approach [53]. Specifically, data within included articles were coded deductively, using the research objectives and primary authors’ (of included articles) interpretations (e.g., theme and category labels) as a framework, as well as inductively, enabling the raw data to inform new codes (that were not guided by research objectives or the primary authors’ interpretations). Following coding, AW and SG met to review their codes and begin the iterative process of reflecting, exploring alternative understandings, and grouping codes together to form subthemes and themes [49]. At this point, for those articles that did not include direct participant quotations (i.e [54–58]), authors’ interpretations were reviewed to explore whether insights emerged that differed from those articles with direct participant quotes; no areas of divergence were noted. After subthemes and themes were finalized, they were grouped into higher-order categories to better organize findings. Of note, during the meta-data analysis, AW and SG also independently, inductively coded recommendations for future research, that were specific to the purpose of this meta-synthesis, within the discussion sections of included articles. All coded data were reviewed and summarized to better clarify gaps in knowledge and to inform recommendations for future research and practice as related to better understanding pediatric cancer patients’ and survivors’ experiences with PA. Throughout meta-data analysis, a third author (JP) served as a ‘critical friend’.

As a final step, once meta-method, meta-theory, and meta-data analyses were complete, 3 authors (AW, SG, JP) reviewed and discussed all summarized data. Through an iterative process, patterns in findings were identified, (possible) influences were explored, and an overarching narrative of pediatric cancer patients’ and survivors’ experiences with PA, including knowledge gaps, was developed.

## Results

### Included articles

**Fig. 1:**
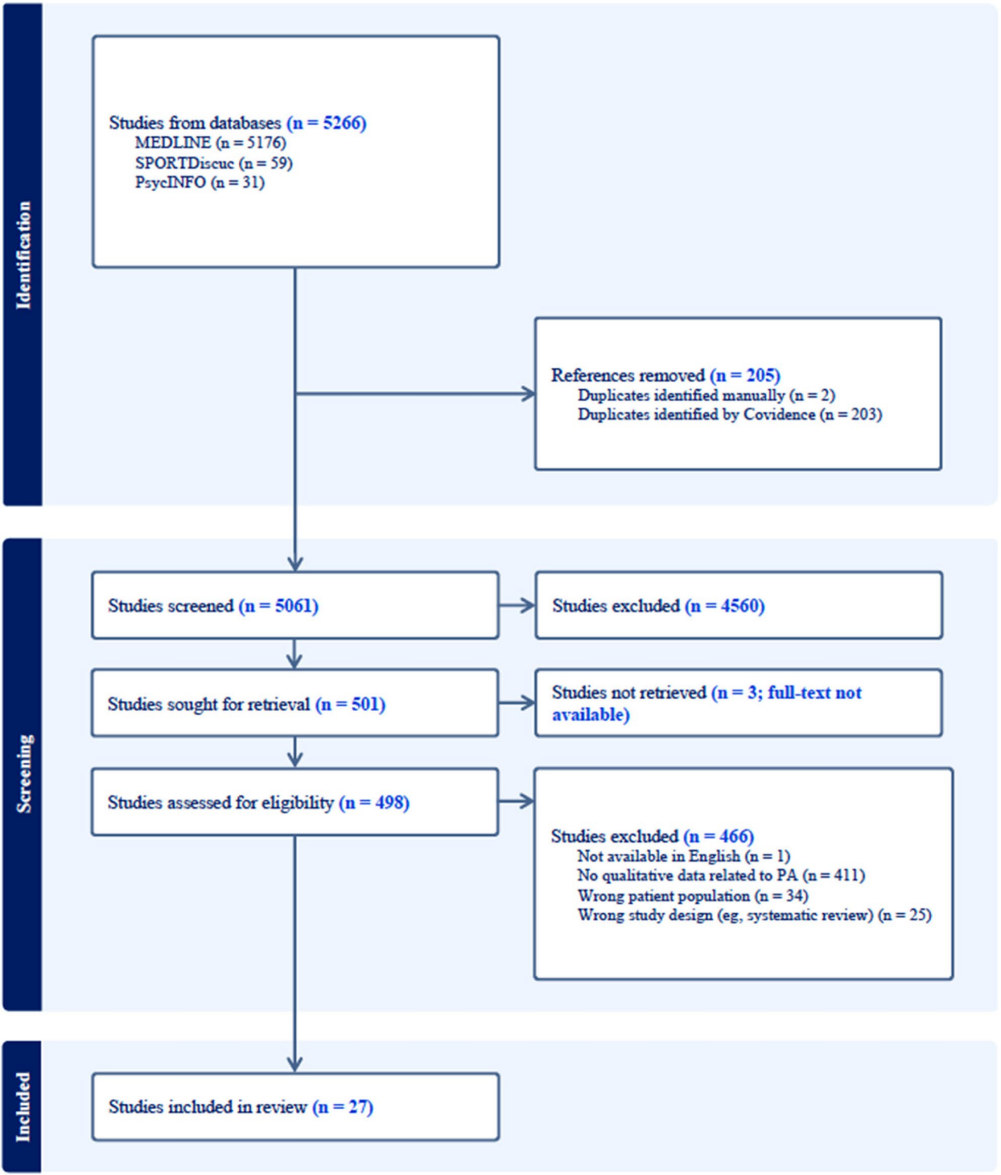
PRISMA diagram

The first database search yielded 4443 references and the second 823 references for a total of 5266 references. Of these, 205 references were identified as duplicates, 5061 unique references were reviewed at the title/abstract level, and 501 full-texts were deemed potentially relevant (inter-rater reliability was moderate at this stage; κ₁ = 0.76; κ₂ = 0.64). Following full-text review, 27 met eligibility criteria [45, 54–79]; the remaining 474 were excluded for the following reasons: did not present qualitative findings related to PA (*n* = 411), did not include pediatric cancer patients or survivors (*n* = 34), not a primary study (*n* = 25), no full-text available (*n* = 3), and not in English (*n* = 1). Inter‑rater reliability was moderate to strong at this stage (κ₁ = 0.8; κ₂ = 0.8). The PRISMA diagram of this process is provided in Fig. 1.

### Reporting completeness assessment

The reporting completeness of included articles, as assessed using the 21-item SRQR checklist, is summarized in Table 1. The *Title and Abstract* domain was generally poorly reported; articles inconsistently made reference to qualitative or mixed-methods in the title (*n* = 8; 29.6%) and abstract (*n* = 18; 66.7%). The *Introduction* domain was well-reported with all (*n* = 27; 100%) articles including their problem formulation and research questions. The *Methods* domain varied in the level of reporting; in this domain, the most reported item was units of study (i.e., participant description; *n* = 26; 96.3%) and the least reported item was researcher characteristics and reflexivity (*n* = 6; 22.2%). The *Results* domain reporting was well-reported with the synthesis and interpretation of findings (*n* = 27; 100%) and links to empirical data (i.e., quotations; *n* = 21; 77.8) relatively consistently incorporated into the articles. The *Discussion* domain was also well-reported with integration with prior work (*n* = 27; 100%) and limitations (*n* = 23; 85.2) generally reported. Finally, the *Other* domain had mixed reporting, with most articles reporting conflicts of interest (*n* = 20; 74.1%) but less than half describing funding sources (*n* = 13; 48.1%).

**Table 1:** Quality assessment of articles included in meta-synthesis (*n* = 27) using the standards for reporting qualitative research checklist

Checklist component	*N*	(%)
*‘Title and Abstract’*		
Title	8	(29.6)
Abstract	18	(66.7)
*‘Introduction’*		
Problem formation	27	(100)
Purpose or research question	27	(100)
*‘Methods’*		
Qualitative approach and research paradigm	7	(25.9)
Researcher characteristics and reflexivity	6	(22.2)
Context	14	(51.9)
Sampling strategy	22	(81.5)
Ethics	24	(88.9)
Data collection methods	24	(88.9)
Data collection instruments	23	(85.2)
Units of study	26	(96.3)
Data processing	16	(59.3)
Data analysis	19	(70.4)
Trustworthiness	7	(25.9)
*‘Results/Findings’*		
Synthesis and interpretation	25	(92.6)
Links to empirical data	21	(77.8)
*‘Discussion’*		
Integration with prior work	27	(100)
Limitations	23	(85.2)
*‘Other’*		
Conflict of interests	20	(74.1)
Funding	13	(48.1)

### Meta-methods results

Characteristics of included articles can be found in Table 2. Briefly, the 27 articles were published between 2009 and 2024 with 14 (51.9%) articles published in the last 5 years (since 2020). Four (14.8%) were conducted in Canada, 6 (22.2%) in China, 5 (18.5%) in the United States, 2 (7.5%) in Germany, 2 (7.4%) in Denmark, 2 (7.4%) in Australia, 1 (3.7%) in England, 1 (3.7%) in Greece, 1 (3.7%) in Finland, 1 (3.7%) in Netherlands, 1 (3.7%) in Norway, and 1 (3.7%) in Norway and Denmark. Twelve (44.4%) articles were purely qualitative studies and 15 (55.6%) were mixed methods. With regards to study design, 14 (51.9%) articles were classified as experimental wherein participants were provided with PA or an opportunity to engage in PA by the *researchers *or* authors* (of included articles) and 13 (48.1%) were classified as observational wherein PA was studied as it occurred in routine practice, usability testing, community programs, or daily life; these articles focused on factors related to PA behaviour (*n* = 11; 84.6%) and cancer-related experiences wherein PA was not the focus of the articles (*n* = 2; 15.4%). Where explanations of designs or studies were unclear, a reasonable level of inference was used to identify the most likely design used.

**Table 2:** Characteristics of studies included in meta-synthesis (*n* = 27)

First author & year	Country	Sample	Study design and approach	Intervention description	Data collection methods	Analysis methods	Theoretical/conceptual orientation
				Experimental			
Chung et al., 2015 [61]	China	*N* = 69 (36 M,33 F); (Sex: NR); Cancer types: leukemia, lymphoma, other; Treatment status: off-treatment; Ages: NR (M = 12.6)	2-group pre/post-test	24 weeks, 4 days Integrated adventure-based training In-person at camp training center, group-based	Interviews	NR	Kolb’s experiential learning theory; Self-efficacy theory; Transtheoretical model
DeNysschen et al., 2021 [62]	United States of America	*N* = 24 (14 M,10 F); Cancer types: NR; Treatment status: off-treatment; Ages: NR (M = 16.6)	1-group pre/post-test	8 weeks, 1 session/week Aerobic and strength training with nutrition education and goal setting Location NR, 1-to-1	Open-ended survey	NR	NR
Diorio et al., 2015 [55]	Canada	*N* = 11 (6 M,5 F); Cancer types: leukemia/lymphoma, solid/brain tumor, aplastic anemia; Treatment status: on-treatment; Ages: 7.7–16.4 (Median: 14)	1-group, pre/post-test pilot trial	3 weeks, 3 sessions/week Yoga In-person in hospital, 1-to-1	Comments	NR	NR
Friedman et al., 2024 [63]	United States of America	*N* = 27 (12 M,15 F) Cancer types: leukemia, neuroblastoma, Wilms, NHL, sarcoma; Treatment status: off-treatment Ages: NR (Median = 14)	Single group pilot	12 weeks, every day Fitbit gamification Home based, social setting NA	Interviews	Rapid qualitative data analysis	Gamification theory
Grimshaw et al., 2024 [65]	Australia	*N* = 20 (11 M,9 F) Cancer types: AML, ALL, Burkitt lymphoma, HL, neuroblastoma, osteosarcoma Treatment status: on-treatment Ages: 9–14 (M = 13)	Single-group, repeated measures, mixed methods design	10 weeks, 1 session/week Fitbit + behaviour support In person in hospital and virtual at home after discharge, 1-to-1	Interviews	Thematic analysis	Behaviour change wheel
Hamari et al., 2019 [56]	Finland	*N* = 36 (26 M,10 F); Cancer types: ALL, Wilms’ tumor, NHL, HL, other; Treatment status: on-treatment; Ages: 3–16 (M = 7.8)	Randomized controlled trial	8 weeks, every day Wii Fit game In-person in hospital and home-based (parental supervision) supported by physical therapist, 1-to-1	Interviews, observations	Qualitative content analysis	NR
Kohler et al., 2024 [67]	Australia	*N* = 6 (5 M,1 F) Cancer types: cerebellar astrocytoma, medulloblastoma, brain stem glioma, ependymoma; Treatment status: off-treatment Ages: NR (M = 10.6)	Randomized controlled trial	12 weeks, 1 session/week Aerobic and activities specific to goals In person in varied locations, 1-to-1 + 3 sessions/week self-directed in home	Interviews	Inductive content analysis	NR
Lam et al., 2020 [69]	China	*N* = 23 (13 M,10 F); Cancer types: leukemia, lymphoma, brain/spinal, germ-cell, bone; Treatment status: on-treatment; Ages: NR (M = 12.4)	Descriptive phenomenological	24 weeks, regressive dosage Integrated experiential training program (aerobic, strength, stretching) In-person in home, 1-to-1	Interviews	Descriptive phenomenological analysis	NR
Nani et al., 2019 [71]	Greece	*N* = 3 (1 M,2 F); Cancer types: AML, retinoblastoma; Treatment status: on-treatment; Ages: 5–6 (M = 5.6)	Qualitative	12 weeks, 3 sessions/week Kinect exergames In-person in hospital, social setting NR	Interviews, observations	Thematic analysis	NR
Stein et al., 2019 [75]	Canada	*N* = 10 (3 M,7 F); Cancer types: solid/brain tumor; leukemia/lymphoma; Treatment status: on-treatment; Ages: 11–18 (Median = 15.1)	Pilot trial	10-weeks, 1 session/week Yoga, 3 sessions in-person in hospital and 9 via Skype, 1-to-1	Qualitative feedback/comments	NR	NR
Stössel et al., 2020 [57]	Germany	*N* = 33 (20 M,13 F); Cancer types: leukemia/T-cell lymphoma, CNS tumor; Treatment status: on-treatment; Ages 4.1–17.7	Randomized controlled trial	6–8 weeks, 3 sessions/week, Endurance, strength, and balance activities In-person in hospital and home, social setting NR	Interviews	NR	NR
Takken et al., 2009 [58]	Netherlands	*N* = 8 (3 M,5 F); Cancer type: ALL; Treatment status: off-treatment; Ages: 6–14 (M = 9.3)	1-group pre/post	12 weeks, 2 sessions/week Phased exercise plan In-person at physiotherapy clinic and home, 1-to-1	Open-ended survey	NR	NR
Thorsteinsson et al., 2019 [76]	Denmark	*N* = 13 (7 M,6 F); Cancer type: ALL, lymphoma, LCH, AML, CNS, solid tumors; Treatment status: mixed on-/off-treatment; Ages: 8–16 (M = 12.23)	Qualitative study embedded in population-based prospective, controlled, mixed‐methods inter‐ vention study	Varied length (treatment dependent), 3–5 sessions/week for individual and 2 sessions/week for group Functionality, aerobic, strength, and stretching activities In-person in hospital, 1-to-1 and group-based	Interview	Strategy systematic text condensation	Self-determination theory; Social cognitive theory
Williamson-Lewis et al., 2023 [77]	United States of America	*N* = 44 (21 M,23 F); leukemia, lymphoma, solid tumor; Treatment status: off-treatment; Ages: NR (M = 15.1)	1 group feasibility trial	52 weeks, every day Fitbit Flex Home based, social setting NA	Open-ended survey	NR	NR
***Observational***							
Bruggers et al., 2018 [59]	United States of America	*N* = 10 (Sex: NR); Cancer types: NR; Treatment status: on-treatment; Ages: NR (M = NR)	Mixed methods usability assessment	1 session Empower Stars! video game, aerobic In-person in hospital, 1-to-1	Open-ended survey	NR	NR
Burke et al., 2017 [80]	England	*N* = 4 (4 M); Cancer types: brain tumor, ALL; Treatment status: off-treatment; Ages: 8–13 (M = 10.3)	Qualitative multiple case study	Duration NA, frequency NA Charity program provides bicycles Home-based, social setting NA	Interviews	Case study methodology	NR
Chung et al., 2014 [54]	China	*N* = 128 (67 M,61 F); Cancer types: leukemia, lymphoma, brain, osteosarcoma, kidney, germ-cell; Treatment status: off-treatment; Ages 9–16 (M = 12.6)	Mixed methods cross-sectional	NA	Open-ended survey	NR	Transtheoretical model of behaviour change
Götte et al., 2014 [64]	Germany	*N* = 40 (21 M,19 F); Cancer types: leukemia (ALL/AML), lymphoma, bone, other solid tumors; Treatment status: on-treatment; Ages: 4–20 (M = 13.2)	Cross-sectional qualitative	Duration NA, frequency NA Supervised exercise In-person in hospital, social setting NR	Interviews	Grounded theory and content analysis	NR
Gu et al., 2014 [66]	China	*N* = 20 (9 M,11 F) Cancer types: All, AML, Osteosarcoma, Brain, Kidney, Lymphomas; Treatment status: off-treatment Ages: NR (M = 9.6)	Descriptive qualitative	NA	Interviews	Thematic analysis	NR
Lam et al., 2017 [68]	China	*N* = 25 (20 M,5 F); Cancer types: leukemia, brain/spinal tumor; Treatment status: on-treatment; Ages: NR (M = 12.6)	Descriptive phenomenological	NA	Interviews	NR	NR
Larsen et al., 2022 [70]	Norway	*N* = 22 (14 M,8 F); Cancer types: ALL, HL, NHL, CNS, solid; Treatment status: off-treatment; Mean age 14.1	Descriptive qualitative	NA	Interviews	Hybrid inductive–deductive thematic analysis	NR
Larsen et al., 2023 [45]	Norway & Denmark	*N* = 63 (39 M,24 F); Cancer types: leukemia, CNS, NHL, HL, solid; Treatment status: off-treatment; Ages: 10–18 (M = 14)	Qualitative	NA	Interviews	Systematic text condensation	International classification of functioning, disability and health, children and youth version
Petersen et al., 2022 [72]	Denmark	*N* = 15 (9 M,6 F); Cancer types: CNS, extracranial solid, lymphoma, leukemia; Treatment status: off-treatment; Ages: 11–18 (M = 14.9)	Explorative, qualitative (phenomenological-hermeneutic)	NA	Interviews	Thematic analysis	NR
Pierzynski et al., 2020 [73]	United States of America	*N* = 51 (20 M,31 F); Cancer types: non-CNS solid, leukemia, CNS, lymphoma; Treatment status: off-treatment; Ages: NR (M = 13.8)	Qualitative	NA	Interviews	NR	NR
Price et al., 2021 [74]	Canada	*N* = 10 (7 M,3 F); Cancer types: leukemia, lymphoma, osteosarcoma, synovial sarcoma, brain; Treatment status: off-treatment; Ages 15–25 (mean 17.4)	Qualitative description, mixed methods	NA	Interviews	Thematic analysis	NR
Wright et al., 2013 [78]	Canada	*N* = 48 (29 M,19 F); Cancer types: leukemia, solid tumor, lymphoma, CNS; Treatment status: off-treatment; Ages: NR (M = 16)	Mixed methods	NA	Interviews	Directive content analysis	NR
Xu et al., 2022 [79]	China	*N* = 35 (19 M,16 F); Cancer types: leukemia/lymphoma; bone, ovarian, brain, neuroblastoma; Treatment status: off-treatment; Ages: 9–17 (M = 12.2)	Qualitative	NA	Interviews	Thematic analysis	Health belief model

*ALL* acute lymphoblastic leukemia, *AML* acute myeloid leukemia, *CNS* central nervous system, *HL* Hodgkin lymphoma, *NHL* Non-Hodgkin lymphoma, *NR* Not reported, *NA* Not applicable, *M* males/boys, *F* females/girls

### Sample characteristics

In total, 798 pediatric participants were included across the 27 articles, with sample sizes ranging from 3 to 128 (*M* = 29.6, SD = 26.5). Twenty-six (96.3%) articles reported the gender/sex of participants, comprising 440 boys/males and 352 girls/females, resulting in an overall girl/female representation of approximately 44.1%. However, the percentage of females/girls in individual studies varied widely, ranging from 0% to 70.0%. The average age of participants ranged from 5.6 to 17.4 years. All articles specified when participants were recruited; 16 (59.3%) included participants beyond treatment, 10 (37.0%) during treatment, and 1 (3.7%) mixed treatment (i.e., during and beyond treatment). Twenty-five (92.6%) articles reported type(s) of cancer; 24 (88.8%) mixed diagnoses and 1 (3.8%) leukemia. Four (14.8%) articles reported recruiting cancer stages 1–4.

### Intervention characteristics

Of the 14 articles that were classified as experimental, the interventions were PA-only (*n* = 7; 50.0%), multimodal (*n* = 2; 14.3%) combining PA with other modalities (e.g., nutrition education, motivational support), active video gaming (*n* = 2; 14.3%), or wearable fitness trackers (*n* = 3; 21.4%). The interventions varied significantly in type, duration, frequency, intensity, session length, setting, supervision, and context. Notably, two of the articles classified as observational recruited from contexts wherein a PA program was available as part of hospital services [64] or a community cycling program [60].

The PA-only interventions included yoga (*n* = 2; 14.3%) and mixed PA interventions (*n* = 5; 35.7%) combining strength, endurance, balance, and flexibility training. The multimodal interventions took different approaches, with one (7.2%) offering adventure-based training with health education and one (7.2%) offering fitness instruction, nutrition education, and goal setting. For the wearable fitness trackers, these were provided on their own (*n* = 1; 7.2%), with a social gamification platform (*n* = 1; 7.2%), or with behavioral support (*n* = 1; 7.2%). The active video gaming interventions utilized gaming consoles.

For intervention duration, lengths varied from 3 weeks (i.e., yoga) to as long as 52 weeks (i.e., wearable fitness trackers). Common durations included short-term programs lasting 3 to 8 weeks (*n* = 4; 28.6%), medium-term programs of 10 to 12 weeks (*n* = 6; 42.9%), and long-term programs extending from 24 weeks to one year (*n* = 3; 21.4%). One (5.4%) article reported varied intervention lengths that were dependent on participant progress or individual treatment schedules.

For session frequency, frequencies ranged from once per week (*n* = 4; 28.6%) to daily sessions (*n* = 3; 21.4%). One (7.1%) intervention had 2 sessions a week for the first 4 weeks and then reduced to 1 session for the remainder of the intervention. While another (7.1%) intervention offered 4 sessions over a 6-month period.

Intensity of activities was specified in 12 (85.7%) articles and included light (*n* = 1; 7.1%), moderate (*n* = 1; 7.1%), light-moderate (*n* = 1; 7.1%), moderate-vigorous (*n* = 1; 7.1%), and light-vigorous (*n* = 3; 21.4%). One (7.1%) article specified a gradual increase in intensity over time and 4 (28.6%) articles reported customizing intensity based on participant tolerance, fatigue levels, and preferences.

Session duration was reported in 10 (71.4%) articles; 6 (42.9%) had pre-determined sessions lengths that ranged from 30 to 60 min, with a median of 45 min. Four (28.6%) interventions had session lengths that were flexible, accommodating energy levels, endurance improvements, and medical schedules.

The settings of interventions varied. Three (21.4%) interventions took place in-person at hospitals, utilizing facilities like hospital gyms equipped with specialized equipment or wards for bedside or ward-based activities for inpatients. Three (21.4%) intervention took place at participants’ homes, 1 (7.1%) intervention was conducted in a community location (i.e., day camp training centre), and 1 (7.1%) was conducted in a location of the participant’s choosing (e.g., research facility, home, public park/pools). Five (35.7%) interventions combined multiple settings, including in-person at hospitals and home (*n* = 3; 21.4%), in-person at physiotherapy clinics and home virtually (*n* = 1; 7.1%), and primarily virtual with 3 in-person in hospital sessions. Finally, 1 (7.1%) intervention was in-person but location details were not reported.

Supervision was reported in 11 (85.7%) articles, featuring supervision by nursing students (*n* = 1; 7.1%), yoga instructors (*n* = 2; 14.3%), researchers (*n* = 3; 21.4%), physical therapists (*n* = 4; 28.6%), and personal trainers (*n* = 1; 5.9%). Regarding the social context, 12 (85.7%) articles reported the social setting of the intervention. Ten (71.4%) were delivered on a one-on-one basis, 1 (7.1%) was group-based, and 1 (7.1%) one-on-one basis and included group activities.

### Epistemology, methodology, and methods

Of the eight (29.6%) articles that specified the qualitative methodology used, 4 (14.8%) cited qualitative description, 3 (11.1%) phenomenology, and 1 (3.7%) case study. Across the 27 articles, 20 (74.1%) used interviews, 5 (18.5%) open-ended survey questions, 2 (7.4%) comments collected during conversations, and 2 (7.4%) a mix of interviews and observations. Qualitative data analysis techniques were reported in 16 (59.3%) articles, techniques included: thematic analysis (*n* = 7; 25.9%), content analysis (*n* = 3; 11.1%), principles of systematic text condensation (*n* = 2; 7.4%), phenomenological (*n* = 1; 3.7%), inductive-deductive case study (*n* = 1; 3.7%), rapid qualitative data analysis (*n* = 1; 3.7%), and a combination of grounded theory and content analysis (*n* = 1; 3.7%).

### Meta-theory results

Seven (25.9%) of the 27 articles reported using theory or models. One used the transtheoretical model of behaviour change to guide study conceptualization, quantitative data collection and analysis, and interpretation of the results [54]. One used self-determination theory and social cognitive theory to guide study conceptualization, intervention development, qualitative data collection, and interpretation of findings [76]. One used the international classification of functioning, disability, and health for children and youths model to guide study conceptualization, qualitative data collection and analysis, and interpretation of the findings [45]. One used the health belief model to guide study conceptualization, qualitative data collection and analysis, and interpretation of the findings [79]. One stated using gamification theory but did not provide a reference to the exact theory used [63]; the theory informed study conceptualization, intervention development, qualitative data collection, and interpretation of findings. One used the behaviour change wheel to inform study conceptualization, intervention development, qualitative data collection, and interpretation of findings [65]. Finally, one used a combination of experiential learning theory, self-efficacy theory, and transtheoretical model of behaviour change to guide study conceptualization, intervention development, quantitative data collection and analysis, and interpretation of the results [61].

### Meta-data results

Across the 27 included articles, codes were grouped into six main themes (with additional subthemes), which were further organized into two overarching categories (denoted in italicized bolding below). While resultant themes and categories represent distinct concepts, some overlap and interaction between themes was evident, reflecting the complex and multifaceted nature of this topic. Themes are each described in greater detail below and an overview of categories, themes and subthemes, and additional supporting quotes to promote transparency are provided in Table 3. Summarized findings related to recommendations are also presented below.

**Table 3:** Illustrative quotations for identified categories, themes, and subthemes

Theme label and description	Subtheme label and description	References	Sample quotations
***Category 1: Physical activity (PA) experiences***			
From what it was to what it is: PA changes in the wake of cancer	--	[45, 60, 61, 64, 68, 70, 72, 73, 76, 78, 79]	“My speed because I used to be really fast and now I am not as fast as I used to be and my legs have become weak, they’ve got weaker.” [60] “It [treatment] makes my muscles not as strong.It’s harder to go on the trampoline. I can’t jump as high now.I used to jump higher than the fence now I can only jump half of the fence.” [60] “I was a member of a football team at school. After cancer treatment, I believed that I was no longer able to join the team because of decreased physical strength and endurance [...].” [61] “I tried to start on the football team again after the treatment, but I was not in good enough physical shape. The others had gone to the football training as usual, so I was physically pretty far behind, so then I just quit.” [45] “Well, just that um by, I guess, by getting tired, it’s harder to move as fast and just be able to keep the speed up to get back, I guess.” [73] “I was so excited that I could move around freely again. I don’t recall facing any difficulties. But I think that I felt a little behind the others when playing football because they had learned a lot while I was away […].”[72] “it was pretty fun to see how well they did [the ambassadors during training sessions] and how much [physical capacity] I had lost because of treatment. It was a bit funny, but also annoying. I was the strongest in class before, and now I’m suddenly the weakest.” [76] “I do not think that treatment change [my exercise behaviours]. …I just do different things.” [78]
The benefits of PA: Enhancing strength, emotional well-being, and connection	The physical benefits	[60, 61, 64, 65, 67–69, 71, 76, 78, 79] [55]	“I reckon my legs have gotten stronger from it [cycling] and I can climb trees again.” [60] “I’ve been out on my bike a lot more and it’s helped me because I’ve got stronger. I was able to do more distance on my bike and even at football it’s making me get better because now I can do two laps of the Astro without stopping.Basically, it just helps me when I go running. It helps me actually like breathe because when I’ve been out on it [bicycle], it’s getting my heart rate up.” [60] “I would say that because I am fitter my body is able to cope with chemotherapy much better.” [64]
			“Sport is getting me fit and what not!” [64] “And you don’t feel weaker and weaker but [rather] that you’re trying to keep a certain level of fitness for your muscles and stuff […].” [76] “My body has become stronger to walk around thousands of meters directly. But initially, I was worn out when walking around 300 m.” [79] “It [PA intervention] just helped me build my muscles and everything and get me stronger, because my muscles used to be really weak and everything.” [65] “Well, I think I’m faster and stronger.” [67] “I saw the other children with cancer having shortness of breath when playing and even walking. However, I can handle to exercise without fainting or shortness of breath. My heart and lung function may be better than the other children in the ward now.” [69] “I feel my body healthy. I felt better […].” [71]
	The emotional benefits	[60–62, 67–71, 75–79] [55]	“Happy because then I am not just stuck inside all day and I’m actually doing some exercise. It makes me worn out but that’s fine because I like it.” [60] “When you have done a cycle you feel like you have achieved something even though you’ve only achieved a couple of miles. Like when you do a 15-mile cycle you feel very proud of yourself, like yeah I did a 15-mile cycle.” [60] “Because once you actually start training…you begin to think… about all the good it’s doing and you feel happier and forget about that medicine you were given that makes you so bad… I think that training puts you in a better mood.” [76]
			“feel happy but also really tired. Your legs hurt the next day… Well, you feel happy that you could do it… that you did something that day…that you can still do something physical.” [76] “I felt so tired and. I did not think I could handle any physical activity at all. However, exercising with the coach makes me feel more competent in exercising because I can try exercising and testing my physical limit safely when the coach is next to me. I understand my physical abilities more.” [69] “ I feel happier…, you are happy when you get married, I got married with the games, (The games) are funny, I love the games, I am happy when I come here, I have fun…, I prefer to be here, I would like to play every day, I’m happy with the games when was coming downstairs (in the room where the Xbox console was located).” [71] “[physical activity] makes you feel better about yourself.” [78] “…Release emotional pressure. After taking PA with sweating, I feel very comfortable.” [79] “I like playing the ball sports, sometimes also take the initiative to go jogging. It seems that I am happy by doing this.” [79]
	The social benefits	[45, 60, 62, 67, 71, 72, 74, 76] [56]	“Yeah I go out all the time […] well before when I didn’t have a bike I didn’t really go out and play with my friends as much because they all had bikes […] Now, they all chase after me [on the bike] because I’m all the way up there and they can’t keep up!” [60] “…working out with my friends and hanging out even while we were exercising.” [62] “I like to get my pulse up. And it is very nice that I play with the boys. I like to play soccer because of my friends include me and win tournaments.” [45] “I think you have to find something you want to do. If you do not know what you want then find something that is fun. You will find friends faster if you sign up for a physical activity.” [72]
			“It’s always fun to train with others who you get al.ong with […] people who you know […] just like last time when we trained […] it was cool that we were able to sit and do those exercises together. I thought that was really cool.” [76] “…it’s always fun to train with others who you get al.ong with…people who you know…just like last time when we trained…it was cool that we were able to sit and do those exercises together. I thought that was really cool.” [76] “I like seeing them [parents] take care of themselves and it also makes them happy as well, like my mom drives to work and works in an office so whenever she will get home, she will say ‘hey [Caleb] do you want to go for a walk or throw a frisbee outside’, which is always pretty great.” [74]
PA interventions: A fun, enjoyable, and appreciated way to engage in PA		[59, 61– 63, 65, 67, 69, 71, 75–77] [55–57]	“Exercise games were fun.” [59] “I enjoyed participating in this special program; the activities were interesting and challenging. Although I needed to make extra efforts to catch up with my peers in academic performance, I did not have to struggle to join the program as it was only organized on separate days at weekends over 6 months.” [61] “I loved this program. It was a great experience and would definitely do it again.” [62]
			“I think…that it was a great idea to have…individualized training because sometimes there’s a need for it instead of group training. It gives … much more focus on you rather than on all the others as well.” [76] “That was real fun, actually. I certainly got a lot from it. I think it was interesting to see that you could actually improve [your physical wellbeing, fitness] during cancer treatment.” [76] “I will probably keep exercising every day. It’s nice when you can walk, it clears your mind. If it’s a morning walk, it’s helpful to kickstart your brain so you’re ready to start the day.” [63]
***Category 2: Influencing factors***			
The burden: Cancer and its treatment	Contextual constraints on movement	[64, 66, 68, 71, 76, 79] [55, 56]	“Getting out of the bed is hard in the first place. When you lie all the time you get accustomed to it very quickly so you really have to pull yourself together to get up.” [64] “I can move around freely because I don’t need to stick with the machine. I can exercise as usual.” [68] “It is so inconvenient to even walk with the device (infusion pump). I can’t be as mobile as before. I can’t exercise.” [68] “It is reduced because I have an infusion port in my body, so like running…my Mom has communicated to my teacher, try to (decrease PA)… if it is not the vigorous PA, I can participate.” [79]
			“I remember going on a few walks even though I had to do it with a drip stand. And I felt that it was totally unfair because it was so boring walking up and down the hospital corridors… especially with that drip stand. Walking back and forth is so boring.” [76] “I think the worst for me here is always lying in my room. That’s why I was pretty happy for the occasional change and the possibility to get out.” [64] “I have a port in my body. I wanted to go swimming, but what if the port gets disconnected? It’s really hard to imagine how scary it would be if it broke (shakes head).” [66]
	The physical cost of treatment	[45, 60, 64, 66, 68, 70, 72, 73, 76, 79] [54, 56]	“I’ve got stretch marks down my legs. Apparently it’s the type of drug, it’s a steroid but whenever I told someone it’s a steroid they’re like ahh so you will be really pumped up but it does the exact opposite of getting you pumped up, it just withers you away to skin and bone pretty much.friends are friends really, they don’t really get it.” [60] “Yeah, and chemotherapy just makes you feel nauseous…and you are totally drained, and I sleep often […].” [64] “t’s hard to go to the gym, when you are so drained, to motivate yourself when you are as tired and exhausted, as I am. Then it is easier to just sleep, listen to music or watch TV, and save the energy.” [45]
			“Actually I don’t have many side-effects from the treatment, just a bit of nausea. And I feel much better in between treatments, so I do much more exercise in that period.” [68] “Without the cancer I would have been higher, stronger and fitter, even stronger than my brother; Now I am like the smallest boy in my class.” [70] “It is very frustrating. I don’t like being this weak. I can’t throw as hard or as far or run as fast, as my friends. My body used all its strength to fight the cancer.” [45] “I stay at home alone, because of this (disease). It is not convenient to go downstairs. Everyone else is at school, and I don’t have anyone to play together…” [79] “I wear a cap when exercising so I look better. Sometimes I exercise indoors too and no other people can see me.” [68] “I’m currently on (chemotherapy) medication, it’s like a storm in my stomach. I don’t dare to get out of bed and move.” [66]
Knowledge, preferences, and beliefs: The PA information held	--	[45, 63, 64, 66, 68– 70, 74, 76, 78, 79] [54, 56]	“Most of the time I know it [physical activity] is good and I value it, but I don’t really think about it.” [74] “Physical activity is important […]. There are other things you need to focus on, like the eating, the fluids, and if in school, focus on your homework, your projects, your tasks and all of that.” [74] “.if you have homework going on or if you have a social event.it’s hard to be physically active.” [78] “When I came home after school, I did not do much exercise. I had to do homework at home.” [79] “I understand that physical activity is good to health in general, like better sleep and appetite. But even if I don’t exercise, I won’t have side-effects like cancer did. So I should now focus on how to be cured from cancer first. I can exercise again after cancer.” [68]
			“I did not feel that it [training at the community center] was my kind of thing, it was very general training.it seemed like they did not think that I with the things that I had, would be able to train myself back into shape. So then I stopped with the physiotherapy […].” [45] “…It gets dark earlier in winter. When I go back home to do my homework, around 5 pm, it is already night, and I cannot play sports outside.” [79] “Walking briskly is my daily activity on the ward. I believe my muscle strength and balance can be regained and improved. If I don’t exercise, my muscles will not be as strong as before and I will also become very fat. That’s why I continue doing these things.” [68] “I like to push myself [laughs], I just think it’s good, and I think it’s good for the body and…. It is, after all, to be in shape, and, there is nothing worse than not being in shape somehow.” [70] “Just before the programme, I really did not put a lot of focus on exercising or learning about its importance. However, now it is very important to me, as I have learnt that exercise can help me reduce the side effects of cancer and treatment.” [69] “Yeah, and it turned out to be a good thing anyway…Yeah, [it’s because] you don’t have the strength… you’re tired or not feeling well enough but when you are pushed to do it, it turns out to be okay anyway. That’s because you’re just happy to be able to train. At least that’s how I see it. So, it’s always nice to be able to train even when you don’t feel up to it at the beginning.” [76]
The role of important others: Support, silence, and control	Healthcare providers	[64, 68, 79]	“They [healthcare provider] told me I wasn’t allowed to play football because of the high risk for injuries. But I should still do something because it’s important. I don’t know […] half an hour exercise a day is good, he said […].”[64] “My doctor did not ask me to exercise. My parents did not suggest me to do so as well because they felt that exercising might make me even more tired from cancer and treatment.” [68] “The doctors said I should exercise more to regain my limb balance and coordination, so that I can regain my ability to move around like I did before cancer and do all the daily activities by myself soon. I want to recover as soon as possible, so I will also do more exercises.” [68] “The doctor told me to do exercises actively. Because, as he said, I would have less muscle and be more fat with fewer exercises.” [79] “I probably wouldn’t have played real ice hockey [if the healthcare provider prohibited ice hockey] then because I was frightened because he (orthopedist) probably had good reasons saying this.” [64]
	Parents and carers	[45, 60, 63, 66, 68, 70, 72, 74, 76–79]	“At the start, we were getting messages about ‘This person didn’t meet their goals; and I would get a chance to yell at my mom and then my mom would get a chance to yell at me. In a positive way.” [63] “Sometimes my dad will go with me for a bike ride, that is support for me. Or when he tells me to do my weights, but he is like there helping me, [but] not actually doing it [lifting weights] with me. There’s those two, both kinds of support.” [74] “[parents].just reminding me.if I haven’t been on the treadmill in a while.” [78] “She [mother] does encourage me to add little things to my day” [74] *“*My mother always asks me to sleep and rest more instead of exercising. She asks me not to move or run, as she worries that I will be exhausted by the treatment if I exercise.” [68] “I asked my mom if I could jump rope, but she refused. I didn’t insist anymore. I must be a special…fragile child.” [66] “Sometimes I don’t bother to monitor [the fatigue]; other times I do. But sometimes I don’t have the strength to monitor myself. That’s when the adults say that I have to go in and rest.” [72]
	Exercise professionals, coaches/trainers, and teachers	[45, 64, 66, 67, 69, 76, 79]	*“*My football coach was very nice [when I got back from treatment]. He said “We put you on the field first, and you play as long as you are able to and then we switch.” [45] “I felt so tired and I did not think I could handle any physical activity at all. However, exercising with the coach makes me feel more competent in exercising because I can try exercising and testing my physical limit safely when the coach is next to me. I understand my physical abilities more.” [69] “Yeah, it was definitely more fun and easier to train when my ambassador was with me.” [76] “My teacher allows me no exercises if I feel tired. They don’t have any requirements on me (in sports).” [79] “I never participated in any sports programs if they were not required by the school.” [79]

Articles [54–58] did not include direct participant quotes and as such are therefore presented separately in the table from the other citations. As described in the main manuscript, in these cases, the authors’ interpretations were reviewed to explore whether insights emerged that differed from those articles with direct participant quotes. No areas of divergence were noted. Further, one article [58], does not appear in this table as authors’ interpretations were unclear with respect to qualitative vs. quantitative data

### Category 1: PA experiences

#### From what it was to what it is: PA changes in the wake of cancer 

This theme captures when participants referred to their changed PA; which often involved expressions of loss, adaptation, frustration, grief, and disappointment. Participants recounted limits to what they could once do; from not being able to jump as high, go as far, move as fast [60, 64, 72, 73], or even “*run like a normal person*” [45]. As one participant shared: “*I went running once or twice…but somehow I just don’t feel like doing it anymore since I won’t manage much distance anyway…I’m just completely exhausted! I’m out of breath!*” [64].

Participants attributed these changes to their diagnosis, treatments, and range of related adverse effects (e.g., pain, shortness of breath, fatigue, stress; [45, 60, 64, 68, 73, 79]; see **Cancer treatment: The context and physical effects below**). For example, one participant shared: “*In the past*,* I usually exercised once or twice a week*,* but since the diagnosis*,* I don’t have any interest in physical activities. I am already so stressed and worried about my disease*” [68]. Some participants indicated they were unable to do the sports and PA they previously enjoyed [61, 70] and that PA that was once fun, no longer was [45]. This prompted some participants to stop engaging in PA because it was defeating [64] and others to reduce or change their PA – either in terms of intensity, overall, or activity types [68, 78, 79].

While many participants described feeling slower or weaker than they once were [45, 60, 64, 68, 70, 73], or left behind in sports and PA [45, 72, 76] (both during and after treatment), it is notable that other participants expressed a sense of excitement and enthusiasm towards PA [45, 72, 76] and felt “*on par*” with their peers, which was captured by one participant who shared: “*I’ve been pretty good at keeping up with the others.I feel like I’m kinda*,* you know*,* on par with everyone else.like keeping it up and doing just as well as the others [...]*” [78].

#### The benefits of PA: Enhancing strength, emotional well-being, and connection

This theme captures when participants recounted the benefits gained from engaging in PA whether generally (i.e., PA in one’s day-to-day life) or as part of a PA intervention. Notably, benefits were described regardless of treatment status (i.e., patient or survivor). Reported benefits spanned domains of physical functioning, emotional well-being, and social opportunities and are represented below as three unique subthemes.

##### The physical benefits

Within this subtheme, participants spoke about the range of physical benefits they acquired through engaging in PA. Specifically, PA was described as helping to decrease aches, pain, and discomfort [60] and improve energy [71, 76] and capacity to cope with cancer-related treatments [64]. For example, one participant shared: “*Well*,* you feel revitalised—really alive and it’s as though you have more energy*” [76]. Participants also recounted noticing improvements in their fitness, strength, and muscle mass [60, 64, 65, 67–69, 71, 78, 79], cardiorespiratory functioning [69], and weight [78]. One participant shared: “*I know I am getting stronger as I do it [PA] and that is a good feeling*” [78]. Participants also shared how PA (and the physical benefits conferred) supported their engagement in other meaningful PA (and other activities; [60, 61, 67, 79]), as illustrated by one participant who shared: “*It [biking] is quite good because it’s also benefitting me in football because I can now kick it quite hard and far*” [60].

##### The emotional benefits

Within this subtheme, participants talked about the emotional benefits they noticed from engaging in PA. Participants described improved mood [60, 68, 71, 76, 78, 79], reduced emotional pressure, such as anxiety and agitation [79], and greater mindfulness/capacity to focus [75]. Even though PA was described as challenging, participants shared a sense of physical competence and confidence [60, 62, 67, 69, 76, 78], accomplishment and pride [60, 76, 77], and mastery [70]. This was captured by one participant who shared: “*I became more confident in myself and more comfortable around others and not worrying what I look like when I work out with others*” [62]. Further, PA conferred feelings of excitement [60] and enjoyment and fun [69, 71, 76]. Notably, PA was also described as instilling a sense of normalcy and control, as expressed by one participant who said: “*Cycling makes me feel happy. When I am doing exercises*,* I feel carefree and normal again*,* even though the activities are not very vigorous. Doing these things helps as long as you enjoy them*” [68].

##### The social benefits

Within this subtheme, participants talked about the social benefits they acquired from engaging in PA. Participants described how PA offered an opportunity to connect with family and peers and provided opportunities to be able to “*hang out*” [45, 60, 62, 67, 74, 76] and to make friends [71, 72]. This was captured by one participant who said: “*It’s more fun to train with others than doing it alone*” [76]. A similar sentiment was captured by another participant who shared: “*I prefer to go hiking with my family. I like when everybody is together*” [45].

#### PA interventions: A fun, enjoyable, and appreciated way to engage in PA 

This theme captures when participants’ perspectives were gathered following engaging in a PA intervention (defined as interventions designed to offer or enhance PA). Overall, PA interventions were appreciated and enjoyed. Participants described the interventions they took part in fun and positive experiences [59, 61, 62, 67, 69, 71, 76] and reported many benefits (described above in **The benefits of physical activity: Enhancing strength, emotional well-being, and connection**). While some participants also shared that they felt challenged during the PA intervention [59, 61, 67, 76], most appreciated being pushed physically [61, 69, 76], as it helped them to reflect on their abilities, and better see the range of improvements and benefits PA conferred [63, 69, 76]. Other valued aspects of PA interventions included: individualising/tailoring PA to match a child’s interests and abilities [67, 75, 76], 1:1 delivery [67], games-based exercise [59, 61, 71], PA education [69], peer [69, 76] and family support [62, 63], self-monitoring (i.e., Fitbits; [63, 65, 77]), scheduling/flexibility that took into account competing (i.e., academic) and other demands [61, 69], trainers/coaches [67, 69, 76], and having home based options [67, 75]; which was captured by one participant who said: “*I loved the Skype sessions because I was in the comfort of my own home*,* so I felt more comfortable doing yoga*” [75]. Notably, many participants described feeling more motivated, like they had built a routine, and committed to continuing with PA following participating in a PA intervention [62, 63, 65, 69, 71]. For example, one participant stated: “*The programme gave me a lot of interesting physical activities and required me to overcome challenges. I had a lot of fun during the process. This made me want to continue exercising regularly*” [69]. Participants also shared recommendations, qualitatively, in one article, suggesting the intervention be longer [62].

### Category 2: influencing factors

#### The burden: Cancer and its treatments

This theme captures when participants described the ways in which their experience with cancer affected their capacity and motivation for PA. Within this theme, the context (i.e., hospital and other treatment-related constraints) and physical impacts of treatment were described.

##### Contextual constraints on movement

This subtheme captures when participants described the ways in which the context/setting they found themselves in during treatment (e.g., the hospital, isolation) influenced their PA. The hospital and isolation were viewed as demotivating, boring, and negatively impacting PA for some participants [64, 66, 68, 76, 79]. This was captured by one participant who stated: “*because one can kill time better when in bed sleeping or watching TV or whatever. It is just way more distracting than being around all the other ill patients; and when one of them throws up I feel sicker myself immediately*” [64]. Other participants spoke of the challenges accessing age-appropriate equipment and movement/rehabilitation services [66] and engaging in PA in the hospital or their hospital room, neither of which were set-up for nor spacious enough to accommodate PA [76]. These sentiments were reflected in quotes by participants who shared: “*Initially we went to the medical institution for rehabilitation*,* but the rehabilitation equipment there was outdated. So*,* we had to transfer to another medical institution*,* but their equipment was also designed for adults…It takes us an hour and a half each day to travel back and forth*” [66] and “…*it’s not so motivating doing exercise in your room*” [76]. Others described how getting accustomed to lying in bed and resting and “waiting for everything to be over and then going home” [64] and missing school, spending less time with friends, and feeling isolated [79] further deterred PA. Others still described how the infusion lines and/or catheters necessary to receive their treatments limited their ability to engage in PA [64, 66, 68, 71, 76, 79] and when they were not hooked up, they could do more [64, 66, 68, 71, 76]. Indeed, one participant shared: “*it is so inconvenient to even walk with the device (infusion pump). I can’t be as mobile as before. I can’t exercise*” [68].

Notably, some participants (who had access to PA during treatment through an intervention or available programming) found that engaging in PA was one way to counteract the negative influence of the context and make the day go faster [64, 76] and provide some variety [76]. This was reflected in a quote from a participant who said: “*Because it [training] makes the day go faster. The main thing when you have cancer is to get rid of it right away…and fast! You don’t feel like having long*,* boring days and feeling bad*” [76].

##### The physical cost of treatment

This subtheme captures when participants shared how cancer, its treatment, and related adverse effects negatively, and often significantly, impacted their ability to engage in PA. Specifically, participants described how treatment made them feel sick and reduced their strength and endurance [45, 60, 66, 70], and caused them to feel weaker [45, 60, 68] in a way that differed from prior to treatment [45, 60, 72] or that was noticeably different from others (e.g., peers; [45]). One participant stated: *“[.] it [treatment] makes my muscles not as strong [...]*” [60]. Participants also described how treatment made their lungs/heart (i.e., chest) feel uncomfortable or different, which deterred them from engaging in PA [79]. Feelings of immense, insurmountable fatigue were commonly shared, which made participants feel too tired for PA or interfered with their capacity to engage in PA [45, 60, 64, 68, 73]. Participants also expressed being fearful of exacerbating their fatigue through PA [68]. One participant said this about how fatigue impacted their PA: “*I always feel limb weakness and tiredness during treatments. Even if I sleep for hours and days*,* the feeling doesn’t go away. I feel tired when standing for a long time. I can’t do exercise and I want to sleep all day*.” [68]. Participants also spoke about the ways nausea and pain [45, 64, 66, 73] reduced their perceived capacity to participate in PA and the ways treatment changed their physical appearance (e.g., hair loss, weight gain), which further deterred their PA [45, 60, 68, 79] so that they could avoid their friends or others from seeing their changed body [68, 79]. One participant elaborated: “*I look so different from normal children now that I have no hair. I don’t want to be labeled as “weird” or as a “cancer victim” when doing outdoor activities. I still feel bad about people knowing that I have cancer*” [68].

To overcome this adverse influence on their PA, some participants described covering up their bodies or engaging in PA in different environments [68]. Others described pushing past feelings of fatigue or being tired (or ignored it) and expressed pride that they were engaging in PA on their own terms [72]. Participants also recommended going slowly and persevering [72], as captured by a participant who shared: “*Take one day at a time. Take it slow. Do the best you can and never give up. Never give up even if it is hard*” [72]. Notably, for those participants who did not experience many side effects, or who felt better between bouts of treatment, cancer, its treatment, and related adverse effects were not a deterrent to PA [68].

#### Knowledge, preferences, and beliefs: The PA information held

This theme captures when participants shared about their knowledge, beliefs, and preferences for PA. With regards to knowledge and beliefs, some participants described the ways their knowledge and beliefs hindered their engagement in PA. For example, one participant shared how their knowledge and beliefs about medicine/healing (e.g., Traditional Chinese Medicine) emphasized rest over PA, therefore deterring PA engagement: “*Chinese medicine emphasizes resting. And I cannot do anything*,*just accept the disease. I prefer sleep rather than exercising*” [68]. Other participants described knowing PA was beneficial and could help them through their cancer experience and improve or maintain their general fitness and health, which motivated them to engage in PA [64, 68, 69, 76, 78, 79]. For example, one participant shared: “*Yes*,* but I also like doing something (exercise) here*,*so you won’t lose your touch completely. So you won’t have to go home in a wheelchair because you feel too weak*” [64]. There were exceptions to this wherein some participants knew PA was good for them, but this knowledge did little to influence their actual behaviour, due to other pertinent and self-identified priorities in their life or feeling self-conscious or as though PA was unachievable [66, 68, 74, 78, 79]. One participant shared: “*Everything proves that it is good for you. There is no reason not to do it*,*but I think there […] are other priorities in life*” [74]. Another stated: “*I saw…They were my classmates*,* and they were skipping rope. I wanted to join them. But I should… I couldn’t. Anyway*,*I was a bit torn*,*so I chose to pretend I didn’t see them*” [66].

Beyond beliefs, some participants described low levels of PA due to their preferences; these participants indicated they simply lacked motivation for PA [64], were disinterested in or disliked PA [45, 68, 79], in some cases because their attention was focused on their disease [68], or were unable to do the PA they wanted because of weather/seasons [79]. For example, one participant stated: “*I didn’t like sports before*,* but I dislike it more now. Not only I am lazy*,* but also I hate doing exercises with my family*,* my parents together*” [79]. Other participants described being motivated by an element of competition [45, 63] and/or the simple fact that, to them, PA was “fun” or enjoyable [64, 70] and just part of who they were and their life [70, 74]. For example, one participant commented: “*It is my lifestyle*,* so it is not really a ‘big thing’ when we go out or are active*,* it is just what we do [as a family]*” [74].

#### The role of important others: support, silence, and control

This theme captures when participants described the role that others played in fostering or hindering their PA. Within this theme, a sentiment of appreciation for support, in general, was shared and healthcare providers, parents (and carers), teachers, and exercise professionals were mentioned as exerting influence.

##### Healthcare providers

Within this subtheme, participants spoke about the influence of healthcare providers. For participants, PA advice from experts was viewed as important – indeed, when healthcare providers gave PA advice, participants indicated they valued and typically followed it [64, 68, 79]. One participant recounted: “*They [healthcare provider] told me I wasn’t allowed to play football because of the high risk for injuries. But I should still do something because it’s important. I don’t know…half an hour exercise a day is good*,* he said…*” [64]. When there was an absence of specific PA recommendations from healthcare providers, PA was less likely to be engaged in [68].

##### Parents and carers

Within this subtheme, participants spoke about the influence of their parents and other carers (e.g., family members), who were viewed as both supporting and hindering their PA. When parents and carers provided instrumental, informational, emotional, and companionship types of support, it was viewed favourably [45, 60, 63, 70, 72, 74] and gentle reminders to engage in PA [78] and friendly competition [60, 63, 77] were appreciated. One participant said: “*My family’s still a good support for me. They are always helping me and making sure I’m doing okay. My dad slows his pace [when out cycling] usually I go ahead of him because then he can make sure I’m there and alright*” [60]. There was also mention of parents exerting force or controlling participants’ PA schedule – interestingly this was viewed as helpful in some cases and harmful in others [45, 74, 76, 79]. This was captured by one participant who shared: “*Sometimes*,* I feel they [parents] are overly supportive since everyday they tell me to do more extra physical activities*” they went on to say “*I want to make the decision of choosing my own physical activities instead of them choosing it for me*” [74]. Finally, when parents expressed worry or concern or asked participants not to engage in PA, it was listened to, and participants did not engage in PA or engaged in less [66, 68], as illustrated by a participant who said: “*My mother always asks me to sleep and rest more instead of exercising. She asks me not to move or run*,* as she worries that I will be exhausted by the treatment if I exercise*” [68].

##### Exercise professionals, coaches/trainers, and teachers

This subtheme captures when participants spoke about the role of exercise professionals, coaches/trainers, and teachers. These individuals were viewed as being in a position to influence PA. For some, exercise professionals and coaches/trainers were viewed as a role model and supported most participants’ PA and PA motivation [64, 67, 69, 76]. One participant shared: “*I faced challenges and failures in the programme. Sometimes*,* I wanted to give up […] She (the coach) stayed beside me when she saw me fail and feel sad. She kept encouraging me and telling me that “failure is the mother of success”. I needed this support to exercise more*” [69]. However, there was one participant who did not appreciate being ‘forced’ to engage in PA. For others, sports coaches provided opportunities to engage in PA [45] and teachers (and schools) were seen as both supporting and hindering PA – through requiring (or not) participation in PA [66, 79]. One participant shared: “*Because I was sick*,* the teachers paid special attention to me. My classmates call me the ‘VIP’ of physical education class*” [66].

### Included articles’ identified gaps and recommendations

All included articles made recommendations for future research, with nearly half underscoring the critical importance of concerted efforts to better support PA and health in this cohort [54, 56, 57, 60–64, 66, 68, 70, 71, 77]. Further, many authors highlighted the ways in which expanding, refining, optimizing, or building upon their intervention or study findings could support PA (and other related outcomes), and ultimately well-being in this population [55–61, 63–67, 69, 73, 75]. Multidisciplinary teams/collaboration were described as essential for advancements [59, 65] and authors commonly suggested avenues for future PA intervention/programming work, including: personalizing/tailoring approaches (based on factors such as age, diagnosis, treatment status, symptoms/side effects, preferences; [54, 58–61, 63, 66, 76], exploring varied PA modalities (e.g., Tai Chi, Qigong; [66], including digital delivery [65, 67, 77] and exergame models [56, 59, 71], complex intervention design that takes into account varied levels of influence [64, 77], including changes to the hospital environment [65, 66] working with community partners to develop PA opportunities [60, 67], ensuring fun, enjoyable, and varied activities [64], offering options within the home environment [67], embedding self-management support [59, 70] and goal-setting [67], considering academic scheduling [58], and creating autonomy supportive environments wherein choice, positive feedback, and safe social interactions are fostered [60]. Finally, the oft-reported necessity of larger sample sizes [57, 61, 64], and longitudinal studies exploring PA perspectives over time [77] and experiences within interventions [63] were also mentioned. Beyond these, 5 additional recommendations, aligned with the overarching purpose of this meta-synthesis, were identified and are listed below.

First, **gathering and centring diverse perspectives, including from important others**, such as parents and carers, healthcare providers, community figures (e.g., teachers), and exercise professionals/coaches was deemed necessary to better understand factors influencing PA and motivation and potential benefits of engaging in PA in this cohort [54, 59, 61, 66, 68, 69, 72, 74, 79]. Relatedly, the importance of co-design and centering diverse perspectives in the intervention developmental process was described as critical [77]. A concerted effort to enhance diversity in samples; from types of cancers diagnosed and cancer stage (i.e., on- or off-treatment) to location (i.e., country) to PA/adherence levels was described as necessary to enable researchers to better compare and contrast findings and better understand possible differences in PA experiences among subsets of the pediatric cancer population [60, 61, 72, 74, 75]. Second,** supporting and including important others within PA interventions** was mentioned as one way to embed social support and ultimately afford the opportunity to better understand important others’ influence on PA and enhance PA among pediatric cancer patients and survivors [58, 62, 64, 66, 67, 70, 72, 74, 76, 78]. Third, **developing PA education for pediatric cancer patients and survivors and parents and carers **to augment PA knowledge and beliefs and minimize fears/worries was indicated as one way to better support PA uptake moving forward [45, 58, 64, 66, 68, 74, 76, 78]. Fourth, **encouraging healthcare providers to promote PA and providing PA supports for healthcare providers** was recommended given their potentially ideal position to discuss and promote PA among their patients [45, 54, 60, 61, 64–66, 68, 72, 79]. Finally, authors of included articles recommended **exploring relevant theories, models, and concepts to better understand the mechanisms underlying the benefits of PA** [45, 54, 57, 60, 74, 79] and **how to facilitate/support PA behaviour change** [63, 65]. Some authors suggested specific theories, models, and concepts including: social cognitive theory and self-efficacy theory [80] suggested by Burke et al. [60], the transtheoretical model of behaviour change [81, 82] suggested by Chung et al. [54], the health belief model [83, 84] suggested by Xu et al. [79], and the World Health Organization International Classification of Functioning – Child and Youth version [85] suggested by Larsen et al. [45]. These theories, models, and concepts were described as supporting intervention development and affording clearer understanding of the benefits of PA and potential pathways through which PA exerts its beneficial effects.

### Overarching narrative

**Fig. 2:**
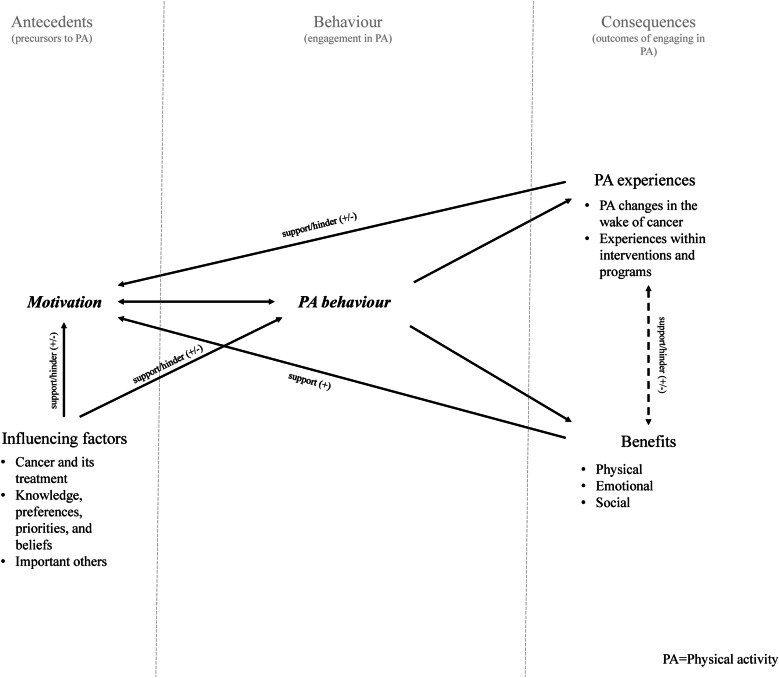
Conceptual model of the overarching narrative of physical activity in pediatric cancer. Note: Unlabelled arrows depict directional relationships that represent processes or sequences wherein one construct gives rise to or informs another. Labelled arrows denote conditional relationships, highlighting that the influence of one construct on another may support or hinder depending on context. Solid arrows represent explicit relationships/pathways and dotted arrows represent latent or inferred understandings

Taken together, findings from this meta-synthesis highlight the nuanced relationships experienced by pediatric cancer patients and survivors between **influencing factors**, **motivation** for and actual **PA behaviour** (latent concepts), and subsequent **PA experiences** and **benefits**. Given limited and inconsistent use of theoretical frameworks across included articles, the A-B-C framework was applied to structure and interpret the relationships identified, offering a coherent lens through which to understand the antecedents (A), behavior (B), and consequences (C) of PA in pediatric cancer. A visual representation of the overarching narrative can be found in Fig. 2.

Influencing factors (i.e., cancer and its treatment; knowledge, preferences, priorities, and beliefs; and, important others) were predominantly drawn from observational studies, which offered in-depth insights into the personal, relational, and contextual dynamics shaping motivation and PA behaviour. Notably, influencing factors were neither inherently positive nor negative, but could support or hinder motivation and PA behaviour, depending on the individual and other contextual factors. For example, while some participants experienced adverse effects from cancer and its treatment which negatively impacted their motivation to engage in PA, others experienced adverse effects, yet were motivated to engage in PA. For these participants, PA was viewed as a way to support them through their cancer experience, ultimately increasing their motivation and PA participation.

Aggregated findings also underscored the complex relationship between pediatric cancer patients’ and survivors’ experiences with PA, including their PA behaviour and benefits accrued. This was generally emphasized in experimental studies foregrounded on measurable or observable improvements tied to specific PA interventions, the exception being, ‘changed PA’ which was drawn from both observational and experimental studies. With respect to the relationship observed, a reciprocal relationship emerged wherein both PA experiences and benefits were described as impacting motivation, the former both positively and negatively and the latter positively. For example, participants described their changed PA and the ways in which this lowered their motivation to engage in PA. Yet, synthesized data suggests this could be countered or modulated through experiencing benefits from PA and/or positive PA experiences.

## Discussion

The overarching purpose of this meta-synthesis was to identify, analyze, synthesize, and interpret qualitative findings related to PA experiences among pediatric cancer patients and survivors. The 27 studies that were included herein, covering pediatric cancer patients and survivors with an average age of 5.6 to 17.4 years, diagnosed with various types of cancer, compellingly demonstrate the varied influences that can shape motivation for and engagement in PA, the complex interplay between cancer and PA experiences, and the range of potential benefits –spanning physical, emotional, and social domains – that PA may confer. Importantly, this meta-synthesis highlights targets for interventions/programs (i.e., influencing factors) and suggests that PA is a promising strategy to support pediatric cancer patients and survivors as they navigate the adverse effects of cancer and its treatments, focus on their health rather than their illness, (re-)discover their strength and physical abilities, feel greater confidence, competence, and normalcy, and foster social connections and support. Overall, synthesizing the personal narratives and lived experiences of pediatric cancer patients and survivors, has generated rich data, in-depth descriptions, and nuanced insights that complement and extend beyond what individual qualitative articles, meta-syntheses, or systematic reviews (e.g., quantitative meta-analyses) have captured to date.

Indeed, the findings from this meta-synthesis re-affirm factors cited in Brown et al.’s [47] recently published meta-synthesis and offer novel insights that address previously noted gaps (including those identified by authors of included articles). For instance, whereas Brown et al. [47] focused exclusively on barriers and facilitators to PA among pediatric cancer survivors, the current synthesis collates perspectives encompassing experiences from both patients and survivors, enriching understanding of PA engagement during and after treatment – of which there were no discernable differences. Additionally, Brown et al. [47] did not identify barriers or facilitators within the Theoretical Domains Framework domains of “knowledge,” “social/professional role and identity,” or “optimism.” In contrast, this meta-synthesis contributes data directly relevant to each of these domains, offering deeper insights into how pediatric cancer patients’ and survivors’ knowledge, beliefs, and preferences shape their motivation and engagement in PA, as well as the role healthcare providers play in communicating the benefits and purpose of PA. It is plausible that the broader focus on overall PA experiences, rather than solely on barriers and facilitators, enabled the identification of factors within these previously unidentified Theoretical Domains Framework domains, offering a more comprehensive understanding of pediatric cancer patients’ and survivors’ PA experiences. Further, beyond barriers and facilitators, this work illuminates dynamic relationships wherein influencing factors, PA experiences, and benefits positively and/or negatively influence motivation, creating a cyclical process that can either support or hinder continued PA engagement depending on individual and contextual factors. This suggests a clear pathway for intervention; by targeting specific influencing factors (e.g., knowledge, preferences, priorities, and beliefs) or PA experiences (e.g., experiences within interventions and programs), motivation and behaviour may be positively reinforced (see Fig. 2). This integrated perspective is not well-developed in pediatric contexts, marking a novel contribution. The lack of consistent theory use described above also points to opportunities for applying behaviour change frameworks, theoretical integration, or utilizing grounded theory approaches to support PA intervention/program design and implementation in pediatric cancer populations.

Findings from this meta-synthesis also complement and align with a previously published meta-synthesis exploring parents’ perceptions of PA for their children with cancer [86], reinforcing the impact of cancer and its treatment on PA experiences. Parents described many of the same influencing factors identified by pediatric patients and survivors, including contextual challenges (e.g., logistical barriers), the role of key social influences (e.g., family support), and individual-level factors (e.g., knowledge, preferences, and beliefs). The need for enhanced support and parental education for PA was also highlighted [86], aligning with recommendations made in the articles included in this meta-synthesis. Taken together, these findings emphasize the need to triangulate diverse perspectives to more fully understand PA experiences and to inform the development of appropriate PA interventions.

Notably, results from this meta-synthesis reinforce, extend, and provide contextual depth to established conclusions from published systematic reviews and meta-analyses exploring PA among pediatric cancer patients and survivors [29–36]. For example, extant literature has documented the physical benefits of PA for pediatric cancer patients and survivors – including improving cardiopulmonary health, functional mobility, muscular strength, body mass index, and reducing fatigue [33, 42, 87]. These conclusions are affirmed herein with participants describing, in detail, the ways their physical health and functioning improved and offering rich context to elucidate the reciprocal relationship between their changed PA (as a result of cancer and its treatment) and observed physical outcomes. Beyond the physical benefits, findings from this meta-synthesis address a critical gap in the quantitative literature, wherein emotional and social effects of PA have been inconsistently cited or subject to mixed or null results (i.e., no effect) (e.g [35, 42]). Findings suggest that PA may serve as a powerful strategy to enhance mood, confidence, and a sense of normalcy, while simultaneously creating meaningful social connections that transcend one’s ‘patient’ identity and strengthen relationships with family and peers. These interconnected physical, emotional, and social benefits align nicely with contemporary conceptualizations of quality of life for those with chronic conditions, including cancer and pediatric cancer, as dynamic, subjective, and interactive [88–91], where experiences in one area (i.e., physical) can catalyze change in another (i.e., emotional). The convergence and extension of synthesized quantitative and qualitative data provides greater support for the integration of PA into pediatric cancer care to support health and well-being, while offering guidance for intervention/program design and implementation strategies.

With respect to offering guidance for intervention/program design and implementation, as described briefly above, findings from this meta-synthesis elucidate key targets to more effectively support PA among pediatric cancer patients and survivors. The overarching narrative, depicted in Fig. 2 highlights the range of influencing factors identified (i.e., cancer and its treatment [contextual constraints, the physical cost of treatment], knowledge, preferences, and beliefs, important others) reinforcing the need to address PA determinants across multiple, interacting levels – including organizational, interpersonal, and individual – and to adopt complex, multi-level approaches to foster PA within this cohort. This aligns with existing evidence and well-established theories, models, and/or theoretical frameworks commonly used to support PA behaviour change (e.g., Capability Opportunity Motivation Behaviour [COM-B]; [92]); however, such theories/models/frameworks have been rarely applied within pediatric exercise oncology literature. In practice, this may look like offering pediatric cancer patients and survivors and parents and carers *education* (alongside the PA intervention) to enhance perceived capability and instill positive knowledge and beliefs about PA, which in turn positively influence motivation and ultimately PA behaviour. As another example, this may include *organizational strategies* to enhance opportunities and improve restrictive treatment spaces within hospitals to better support PA. Though these targets and practical examples are perhaps unsurprising given what is known in the broader behaviour change literature, what is novel, is that Fig. 2 and findings herein are grounded directly in the voices and lived experiences of children with cancer, providing patient-centred insights to inform intervention/program development and implementation.

Importantly, findings also draw attention to the potentially reinforcing or discouraging role of PA experiences themselves, highlighting an additional, actionable target for those seeking to support PA in this cohort. Findings from this meta-synthesis suggest that a child’s experiences with PA drives their subsequent behaviour. Creating autonomy-supportive environments focused on fun, ensuring tailored, individualized, and age-appropriate approaches, and integrating opportunities for children to reflect on and notice their progress may enhance the likelihood of positive PA experiences, which in turn may foster greater motivation for PA and sustained PA behaviour. In practice this may look like using wearable fitness trackers to allow children to self-manage and monitor their progress, providing structured feedback following assessment, and highlighting progressions made in structured exercise sessions. As suggested by authors of included articles, looking ahead, researchers and program developers may want to also consider digital and exergaming solutions, which have been described as engaging, motivating and useful for self-reflection [93]. There is also opportunity to explore the upper limits of PA with children; creating a safe space to explore higher intensity activities that have a normal place in childhood such as jumping, hopping, and running and that are perceived as challenging may support greater self-confidence. Indeed, within this meta-synthesis, children expressed appreciation for being challenged during PA, and could articulate that even when PA was difficult, the benefits were worth it.

As described briefly above in the **Overarching Narrative**, this meta-synthesis confirms that observational and experimental traditions each illuminate only part of the PA puzzle in pediatric oncology. The former captures the layered, lived realities that determine whether, how, and why children move (i.e., influencing factors), whereas the latter foreground measurable (i.e., quantitative) endpoints such as fitness, fatigue, or quality of life. A notable exception was changed PA, which followed from both observational and experimental designs. This suggests the literature may be implicitly shaped by study design and highlights the value of integrating both approaches to more fully understand “how” and “why” PA is taken up – and what it affords – within pediatric cancer contexts. Across the articles, four recurrent methodological limitations were observed. First, there was limited application of theory or reporting of behaviour change techniques/support to guide intervention development and evaluation, making replication difficult. Second, qualitative work was thin and uneven (and poorly reported), with many studies relying on open-ended survey items or brief “exit” interviews that provide limited insight into nuanced concepts such as meaning, identity, and motivation. Third, there was no documented meaningful patient-family involvement in shaping research questions, measures, or intervention content; participatory principles, frameworks, or reporting guidelines (e.g., GRIPP2; [94]) were simply absent from the included articles. Fourth, methodological approaches tailored to complex, multi-component interventions (e.g., hybrid effectiveness-implementation trials), nuanced individual data (e.g., visual analysis), or comparative/adaptive designs (e.g., vigorous intensity options) were not observed. From a methodological perspective, future research could benefit from embedding robust theory from the outset, incorporating genuine co-design processes, selecting study designs that align with intervention complexity, and offering tiered activity options that acknowledge both medical constraints and children’s appetite for challenge. Greater attention to these methodological considerations could shift the conversation from whether PA “works” to for whom, under what conditions, and through which mechanisms it yields meaningful, sustainable benefit.

Taken together, this meta-synthesis contributes essential evidence affirming the role of PA in pediatric cancer and is marked by several notable strengths, including the comprehensive and systematic meta-synthesis approach adopted. The iterative development of the search strategy to ensure sensitivity and specificity, while also remaining broad to enhance the likelihood of varied methodological approaches and types of evidence (including PA in diverse settings; e.g., online, in-person, videogame) across the cancer continuum and from participants with different diagnoses and backgrounds, is also a strength, and enhances the likelihood of robust conclusions that are not influenced by any specific methodological orientation, study design, or medical focus. Finally, the independent involvement of multiple authors through screening, extraction, analysis, and interpretation supports credibility of the synthesized findings.

Notwithstanding the contributions made and inherent strengths of this work, there are notable limitations. First, across included articles, there were latent, underlying assumptions that PA levels were low though this was not directly assessed within qualitative contexts. Thus, it is unclear if/how influencing factors and motivation directly influence actual PA behaviour. Exploring and understanding PA levels and how perceptions differ across different levels of engagement will be important. For example, Lam et al. [68] were able to stratify their sample based on activity level (i.e., high PA and low PA), and found differences between those who were and were not active, which could provide deeper insights into to how to better support PA among those who need it. Second, though reporting completeness of included articles, overall, was relatively high, some included articles provided no/limited qualitative data (e.g., participant quotes). This hindered the richness of insights that could be extracted and integrated into the meta-synthesis, particularly when compared to studies that offered comprehensive and extensive direct quotations. Although authors’ interpretations were evaluated for concordance (in cases with no qualitative data) with the themes developed, the absence of participant voices in these articles represents a significant limitation and greater efforts to include and center participant perspectives (i.e., quotes) is required to ensure transparency and trustworthiness. To this end, readers are reminded the quality and comprehensiveness of this meta-synthesis is fundamentally constrained by the quality of its constituent articles. Third, it is possible that the search strategy developed and used may not have been broad enough, with respect to PA, to identify all relevant articles qualitatively exploring pediatric cancer patients and survivors experience with movement. For example, including the term “play” may have identified articles exploring younger children’s experiences with PA. Relatedly, relevant articles, particularly those recently published but not yet indexed in the searched databases, were inadvertently excluded. As a result, some emerging evidence may not have been captured (e.g [95, 96]), which could have provided additional insights to further inform the findings of this meta-synthesis. Future synthesis efforts should occur at regular intervals as further qualitative work is published. Fourth, while this meta-synthesis sought to include studies with pediatric cancer patient and survivors < 19 years, the average age of participants was 5.6 to 17.4 years, representing school-aged children and adolescents. Consequently, the experiences of younger children are notably absent. This highlights a gap in the literature and may be due in part to the methodological challenges in engaging very young children in qualitative research. Future work could consider inclusion criteria that would afford insights into younger children’s experience via observations or parental perspectives. Fifth, one author independently appraised trustworthiness and theoretical and practical considerations for each study. While a second author/appraiser could have offered an additional interpretive lens, depth of engagement and contextual understanding was prioritized over consensus, consistent with the epistemological stance adopted. Sixth, only peer-reviewed, studies in English were included herein, which could exacerbate the risk of publication bias and limit understanding of pediatric cancer patients’ and survivors’ experiences with PA in other settings. Lastly, despite independent involvement of multiple authors, it is plausible that the authors’ personal and professional backgrounds and biases may have influenced interpretation.

## Conclusions

This review employed robust methods to integrate qualitative research findings and represents a much-needed synthesis of an accumulating body of literature. From the 27 articles reviewed, 2 overarching categories were identified: (i) PA experiences and (ii) influencing factors; that collectively highlight the varied influences that can shape motivation for and engagement in PA, the complex interplay between cancer and PA experiences, and the range of potential benefits – spanning physical, emotional, and social domains – that PA may confer. Ultimately, findings make a valuable contribution to the field of pediatric exercise oncology and elucidate pediatric cancer patients’ and survivors’ experiences with PA, integrate practical insights to support participation, and inform new directions for future research and clinical care.

## Supplementary Information

Supplementary Material 1.

## Data Availability

No datasets were generated or analysed during the current study.

## Abbreviations

COM-B: Capability, opportunity, motivation, behaviour
ENTREQ: Enhancing transparency in reporting the synthesis of qualitative research
MEDLINE: Medical literature analysis and retrieval system online
PA: Physical activity
SRQR: Standards for Reporting Qualitative Research

## Footnotes

1 Objectives stated here differ slightly from those in the prospectively registered PROSPERO protocol (CRD42023432809); edits herein were made to enhance focus and clarity within this manuscript, as such differences are minor and semantic in nature.
