# Supplementary material for: Exploring physical activity experiences among children and adolescents during and beyond cancer treatment: a meta-synthesis of qualitative research
Source: BMC Cancer. 2026 Feb 2;26:326. doi: 10.1186/s12885-025-15490-1 (PMC12973550; doi:10.1186/s12885-025-15490-1)
Supplement: Supplementary file 1 — Supplementary Material 1. [file 12885_2025_15490_MOESM1_ESM.docx]

Supplementary File 1: Database Search Strategies

Ovid MEDLINE(R) and Epub Ahead of Print, In-Process & Other Non-Indexed Citations and Daily

| 1. exp Exercise/ |  |
| --- | --- |
| 2. exercis*.ti,ab,kw. |  |
| 3. exp Exercise Therapy/ |  |
| 4. (exercis* adj1 therap*).ti,ab,kw. |  |
| 5. Sports/ |  |
| 6. sport$3.ti,ab,kw. |  |
| 7. exp Yoga/ |  |
| 8. yoga*.ti,ab,kw. |  |
| 9. ((physical or aerobic or endurance or strength or resistance) adj1 (activity* or activities* or activity-based* or train* or fitness or condition or conditioning)).ti,ab,kw. |  |
| 10. or/1-9 |  |
| 11. exp Neoplasms/ |  |
| 12. (neoplas* or oncolog* or cancer* or tumo?r or oncolog*).ti,ab,kw. |  |
| 13. or/11-12 |  |
| 14. exp Child/ |  |
| 15. (child or children or children* or childhood or kid or kids or young* or juvenile or pediatric or pediatrics or paediatric or paediatrics).ti,ab,kw. |  |
| 16. exp Adolescent/ |  |
| 17. (adolescen* or teen*).ti,ab,kw. |  |
| 18. or/14-17 |  |
| 19. 10 and 13 and 18 |  |
| 20. limit 19 to (english language and yr="2000-Current") |  |
|  |  |
|  |  |
|  |  |
|  |  |

| PsycINFO   \| 1. exp Exercise/ \|  \| \| --- \| --- \| \| 2. exp Physical Activity/ or Aerobic Exercise/ \|  \| \| 3. TI (exercis*) or AB (exercis*) or KW (exercis*) \|  \| \| 4. exp Exercise Therapy/ \|  \| \| 5. TI (exercis* N1 therap*) OR AB (exercis* N1 therap*) OR KW (exercis* N1 therap*) \|  \| \| 6. Sports/ \|  \| \| 7. TI (sport$3) or AB (sport$3) or KW (sport3) \|  \| \| 8. exp Yoga/ \|  \| \| 9. TI yoga* or AB yoga* or KW yoga* \|  \| \| 10. TI ((physical or aerobic or endurance or strength or resistance) N1 (activity* or activities* or activity-based* or train* or fitness or condition or conditioning)) OR AB ((physical or aerobic or endurance or strength or resistance) N1 (activity* or activities* or activity-based* or train* or fitness or condition or conditioning)) OR KW ((physical or aerobic or endurance or strength or resistance) N1 (activity* or activities* or activity-based* or train* or fitness or condition or conditioning)) \|  \| \| 11. or/1-10 \|  \| \| 12. exp Neoplasms/ or exp Oncology/ \|  \| \| 13. TI (neoplas* or oncolog* or cancer* or tumo?r) or AB (neoplas* or oncolog* or cancer* or tumo?r) or KW (neoplas* or oncolog* or cancer* or tumo?r) \|  \| \| 14. TI (oncolog*) or AB (oncolog*) or KW (oncolog*) \|  \| \| 15. or/12-14 \|  \| \| 16. exp Child/ \|  \| \| 17. TI (child or children or children* or childhood or kid or kids or young* or juvenile or pediatric or pediatrics or paediatric or paediatrics) or AB (child or children or children* or childhood or kid or kids or young* or juvenile or pediatric or pediatrics or paediatric or paediatrics) or KW (child or children or children* or childhood or kid or kids or young* or juvenile or pediatric or pediatrics or paediatric or paediatrics) \|  \| \| 18. exp Adolescent/ \|  \| \| 19. TI (adolescen* or teen*) or AB (adolescen* or teen*) or KW (adolescen* or teen*) \|  \| \| 20. or/16-19 \|  \| \| 21. 11 and 15 and 20 \|  \| \| 22. limit 21 to (english language and yr="2000-Current") \|  \| |  |
| --- | --- | --- | --- | --- | --- | --- | --- | --- | --- | --- | --- | --- | --- | --- | --- | --- | --- | --- | --- | --- | --- | --- | --- | --- | --- | --- | --- | --- | --- | --- | --- | --- | --- | --- | --- | --- | --- | --- | --- | --- | --- | --- | --- | --- | --- |

| SPORTDiscus |  |
| --- | --- |

| S14 | S5 AND S9 AND S13 |  |
| --- | --- | --- |
| S13 | S10 OR S11 OR S12 |  |
| S12 | TI ( (child OR children OR children* OR childhood or kid OR kids OR young* OR juvenile OR pediatric OR pediatrics OR paediatric OR paediatrics OR adolescen* OR teen*) ) OR AB ( (child OR children OR children* OR childhood or kid OR kids OR young* OR juvenile OR pediatric OR pediatrics OR paediatric OR paediatrics OR adolescen* OR teen*) ) OR KW ( (child OR children OR children* OR childhood or kid OR kids OR young* OR juvenile OR pediatric OR pediatrics OR paediatric OR paediatrics OR adolescen* OR teen*) ) |  |
| S11 | DE “CHILD" |  |
| S10 | DE “YOUTH” OR DE “TEENAGERS” |  |
| S9 | S6 OR S7 OR S8 |  |
| S8 | DE “CANCER patients” |  |
| S7 | DE "CANCER" |  |
| S6 | TI ( (neoplas* OR oncolog* OR cancer* OR tumo#r) ) OR AB ( (neoplas* OR oncolog* OR cancer* OR tumo#r) ) OR KW ( (neoplas* OR oncolog* OR cancer* OR tumo#r) ) |  |
| S5 | S1 OR S2 OR S3 OR S4 |  |
| S4 | (TI (exercis* OR sport* OR yoga*)) OR (AB (exercis* OR sport* OR yoga*)) OR (KW (exercis* OR sport* OR yoga*)) |  |
| S3 | (( TI ( physical* OR aerobic* OR endurance* OR strength* OR resistance* OR muscl* OR muscul*) N2 (activity* OR activities* OR activity-based* OR train* OR fitness OR condition OR conditioning)) OR (( AB ( physical* OR aerobic* OR endurance* OR strength* OR resistance* OR muscl* OR muscul*) N2 (activity* OR activities* OR activity-based* OR train* OR fitness OR condition OR conditioning)) OR (( KW ( physical* OR aerobic* OR endurance* OR strength* OR resistance* OR muscl* OR muscul*) N2 (activity* OR activities* OR activity-based* OR train* OR fitness OR condition OR conditioning)) |  |
| S2 | DE "ANAEROBIC exercises" OR DE "STRENGTH training" OR DE "MUSCLE strength" OR DE "CIRCUIT training" OR DE "PHYSICAL training & conditioning" OR DE "INTERVAL training" |  |
| S1 | DE "EXERCISE" OR DE “PHYSICAL activity” OR DE “SPORTS” OR DE “YOGA” OR DE "AEROBIC exercises" |  |
